# Analysis of a Panel of 48 Cytokines in BAL Fluids Specifically Identifies IL-8 Levels as the Only Cytokine that Distinguishes Controlled Asthma from Uncontrolled Asthma, and Correlates Inversely with FEV_1_

**DOI:** 10.1371/journal.pone.0126035

**Published:** 2015-05-26

**Authors:** Koa Hosoki, Sun Ying, Christopher Corrigan, Huibin Qi, Alexander Kurosky, Kristofer Jennings, Qian Sun, Istvan Boldogh, Sanjiv Sur

**Affiliations:** 1 Department of Internal Medicine, Division of Allergy and Immunology, University of Texas Medical Branch, Galveston, Texas 77555, United States of America; 2 Division of Asthma, Allergy & Lung Biolog and MRC & Asthma UK Centre in Allergic Mechanisms of Asthma at King's College London, London, SE1, 9RT, United Kingdom; 3 Department of Biochemistry and Molecular Biology, University of Texas Medical Branch, Galveston, Texas 77555, United States of America; 4 Office of Biostatistics, Preventive Medicine and Community Health, University of Texas Medical Branch, Galveston, Texas 77555, United States of America; 5 Department of Microbiology and Immunology, University of Texas Medical Branch, Galveston, Texas 77555, United States of America; Université Libre de Bruxelles, BELGIUM

## Abstract

We sought to identify cells and cytokines in bronchoalveolar lavage (BAL) fluids that distinguish asthma from healthy control subjects and those that distinguish controlled asthma from uncontrolled asthma. Following informed consent, 36 human subjects were recruited for this study. These included 11 healthy control subjects, 15 subjects with controlled asthma with FEV_1_≥80% predicted and 10 subjects with uncontrolled asthma with FEV_1_ <80% predicted. BAL fluid was obtained from all subjects. The numbers of different cell types and the levels of 48 cytokines were measured in these fluids. Compared to healthy control subjects, patients with asthma had significantly more percentages of eosinophils and neutrophils, IL-1RA, IL-1α, IL-1β, IL-2Rα, IL-5, IL-6, IL-7, IL-8, G-CSF, GROα (CXCL1), MIP-1β (CCL4), MIG (CXCL9), RANTES (CCL5) and TRAIL in their BAL fluids. The only inflammatory markers that distinguished controlled asthma from uncontrolled asthma were neutrophil percentage and IL-8 levels, and both were inversely correlated with FEV_1_. We examined whether grouping asthma subjects on the basis of BAL eosinophil % or neutrophil % could identify specific cytokine profiles. The only differences between neutrophil-normal asthma (neutrophil≤2.4%) and neutrophil-high asthma (neutrophils%>2.4%) were a higher BAL fluid IL-8 levels, and a lower FEV_1_ in the latter group. By contrast, compared to eosinophil-normal asthma (eosinophils≤0.3%), eosinophil-high asthma (eosinophils>0.3%) had higher levels of IL-5, IL-13, IL-16, and PDGF-bb, but same neutrophil percentage, IL-8, and FEV_1_. Our results identify neutrophils and IL-8 are the only inflammatory components in BAL fluids that distinguish controlled asthma from uncontrolled asthma, and both correlate inversely with FEV_1_.

## Introduction

Asthma is a complex chronic inflammatory disorder of the airways with a high prevalence rate of approximately 300 million people worldwide [1]. Severe asthma represents approximately 5 to 10% of all subjects with asthma [2], but accounts for 40% of the total cost for asthma care [2] and 30–50% of asthma morbidity [3]. The National Heart, Lung, and Blood Institute’s Severe Asthma Research Program (SARP) demonstrated that reduced FEV_1_ (forced expiratory volume in 1 second), history of pneumonia, and fewer positive skin tests for environmental allergens were critical independent risk factors for severe asthma [4]. However, the SARP study also reported that the well-established biomarkers of asthma, such as blood eosinophils, serum IgE, and exhaled nitric oxide levels, do not differentiate asthma severity or correlate with FEV_1_ or asthma severity [4]. The RET/ATS guidelines have been changed for defining asthma severity to controlled and uncontrolled asthma. It is important to identify specific cytokines that distinguish uncontrolled asthma from controlled asthma in the new guideline to develop novel therapeutic targets for severe asthma.

Increasing evidence suggests that inflammatory cells in the airways can distinguish severe asthma from mild asthma [5–12]. Because sputum samples are collected non-invasively, several studies have evaluated sputum samples, and reported higher percentages of neutrophils in the sputum in severe compared to mild asthma [7, 8]. However, because sputum neutrophil numbers do not correlate with the cell numbers in bronchoalveolar lavage (BAL) fluids from the same subjects [13], it is important to validate the observations of neutrophilia in the sputum by sampling other compartments of the airways. A study of tracheal aspirates from patients intubated for acute severe asthma reported a higher percentage of neutrophils compared to a control group of patients undergoing nonpulmonary surgical procedures [9]. In another study, patients intubated for status asthmaticus exhibited a higher mean percentage of neutrophils in their BAL fluid compared to that from patients with stable mild asthma [10]. We have reported that unlike classic slow-onset progressive fatal asthma, peribronchial lung tissues in sudden-onset fatal asthma had considerably more neutrophils than eosinophils [6]. Thus an increasing body of literature supports the idea that there is an abundance of neutrophils in severe asthma.

Many cytokines and chemokines could theoretically be associated with "neutrophil- rich" and “eosinophil-rich” endotypes of asthma [14]. However, most studies have utilized a candidate cytokine approach to quantify specific cytokines in asthma [9–12]. One such candidate-cytokine study evaluated sputum concentrations of IL-8, and reported higher levels in severe vs. mild asthma [7]. Another study evaluated IL-8 in tracheal aspirates, and reported higher levels in patients intubated for acute severe asthma compared to a control group of patients undergoing surgical procedures unrelated to the lung [9]. Likewise, the concentration of IL-8 in BAL fluid from patients intubated for status asthmaticus was elevated compared to mild asthma [10]. To our knowledge, only two study evaluated an array of over 20 cytokines and chemokines in BAL fluid to identify cytokines that distinguish severe asthma from mild or moderate asthma [15, 16]. One of these studies reported identically level of IL-8 in moderate and severe asthma in children compared to adult controls [15], whereas the other reported no difference in BAL fluid levels of IL-8 between mild asthma and severe asthma [16]. To address this difference in the observations reported in candidate-cytokine studies [9–12] vs. panel-cytokine study [16], we examined a panel of 48 cytokines and chemokines in BAL fluids from healthy control subjects and subjects with controlled and uncontrolled asthma.

## Materials and Methods

### Subjects

Subjects were recruited in the Department of Asthma, Allergy and Lung Biology, King’s College London School of Medicine, U.K. The study was approved by the Ethics Committee of King’s College Hospital, and each participant provided written informed consent. Subjects with asthma were included on the basis of history and a demonstrated reversible airflow limitation (20% variability in forced expiratory volume in one second [FEV_1_] or peak expiratory flow rate), increased airway responsiveness to methacholine (concentration producing a decrease of 20% from base line in FEV_1_ [PC_20_], < 8 mg per millilitre), or both. None had ever smoked, and there was no history of other respiratory disease. Atopy was defined as the presence of one or more positive skin prick tests to a range of common aeroallergens. The normal controls had no history of allergic disease, had normal FEV_1_, and a PC_20_ of more than 32 mg per millilitre. Of the controls, 5 of 11 were atopic. The subjects’ characteristics are shown in Table 1. These included 11 healthy control subjects (FEV_1_ = 102%, 89–110), 15 subjects with controlled asthma (Mean FEV_1_ 98%, 81–113) and 10 with uncontrolled asthma (Mean FEV_1_ 64%, 48–74, < 80%). For the purpose of this study, we defined asthma severity based on FEV_1_ while on treatment, according to international ERS/ATS guidelines [17].

**Table 1:** Patient characteristics.

Characteristic	Healthy	Controlled Asthma	Uncontrolled Asthma
n	11	15	10
Age (yr)	24.0 (19–38)	27.1 (19–41)	45.8 (29–63)* ^+^
Sex (% male)	45.5	40	60
Use of ICS (%)	none	11.7	100
Mean dose of ICS	none	27	710
Use of LABA (%)	none	none	90
Duration of asthma, (yr)	NA	4.7 (3–13)	10.0 (6–20)
Atopy, %	54.5	66.7	70
FEV1, % predicted	102.0 (89–118)	98.1 (83–113)	64.1 (48–74) * ^+^
Total IgE (IU/ml)	49.6 (17–232)	86.7 (19–623) *	123.2 (18–721) *
Blood eosinophils (%leukocytes)	0.6 (0.2–2.6)	2.1 (0.6–3.2) *	2.9 (0.4–4.1) *

Results expressed in means and range.

*Statistical significant compared to healthy group

^+^Statistical significant compared to controlled asthma

### Fiberoptic bronchoscopy and collection of BAL fluid

Fiberoptic bronchoscopy was performed, and BAL fluid obtained and processed as previously described [18]. Briefly, bronchoscopy was performed by the same operator in both the asthmatics and the controls after they had received 2.5 mg of albuterol by nebulizer, 0.6 mg of atropine, midazolam for sedation, and 2% or 4% of lidocaine for local anaesthesia. BAL was performed by instilling four 60-ml aliquots of warmed, pH-adjusted, normal saline into either the right middle lobe or the lingula. After collection, BAL cells were centrifuged at 300 x g for 7 min, washed once, and resuspended in 1.5 mL of PBS; BAL fluid supernatants were distributed into 10 ml each tube and stored at -80°C for further analysis (up to 3 years). The mean total amount of BAL fluid was 92ml.

### Cell counts in BAL fluid

Cytospin slides of BAL cells were made with a Shandon 2 cytospin device (Shandon Southern Instruments, Runcorn, UK). For cell differentiation, slides were stained with May-Grunwald Giemsa. Cell counts were performed and the absolute numbers and percentages of eosinophils, neutrophils, lymphocytes and monocytes/macrophages were quantified.

### Cytokines and chemokines in BAL fluid

Cytokines in BAL fluid were quantified using a Bio-Plex array for 48 cytokines (Bio-Rad, Hercules, CA) according to the manufacturer’s instructions: Interleukin (IL)-1α, IL-1β, IL-1 receptor antagonist (IL-1RA), IL-2, IL-2Rα, IL-3, IL-4, IL-5, IL-6, IL-7, IL-8, IL-9, IL-10, IL-12 (p40), IL-12 (p70), IL-13, IL-15, IL-16, IL-17, IL-18, fibroblast growth factor (FGF), eotaxin (CCL11), granulocyte colony-stimulating factor (G-CSF), granulocyte-macrophage colony-stimulating factor (GM-CSF), interferon (IFN)-γ, interferon gamma-induced protein (IP)-10/CXCL10, monocyte chemotactic protein (MCP)-1/CCL2, macrophage inflammatory protein (MIP)-1α/CCL3, MIP-1β/CCL4, platelet-derived growth factor (PDGF), regulated-on-activation normal T-cell expressed and secreted (RANTES)/CCL5, tumor necrosis factor (TNF)-α, vascular endothelial growth factor (VEGF), cutaneous T cell attracting chemokine (CTACK)/ CCL27, growth regulated oncogene α (GROα)/ CXCL1, hepatocyte growth factor (HGF), IFN-α2, leukemia inhibitory factor (LIF), MCP-3/CCL7, macrophage colony-stimulating factor (M-CSF), macrophage migration inhibitory factor (MIF), monokine induced by interferon-gamma (MIG)/CXCL9, nerve growth factor-β (NGF-β), stem cell factor (SCF), stem cell growth factor-β (SCGF-β), stromal cell-derived factor-1α (SDF-1α), TNF-β, and TNF-related-apoptosis-induced- ligand (TRAIL). The lower limits of detection of cytokines that were not detected (ND) or were at borderline limits of detection were: IL-2 (3 pg/ml), IL-4 (6 pg/ml), IL-13 (4 pg/ml), IL-17 (48 pg/ml), FGF (172.4pg/ml), CCL3 (178 pg/ml), CCL11 (325 pg/ml), GM-CSF (22 pg/ml), and TNF-α (543 pg/ml).

### Statistical Analysis

The results of the study are presented as means ± SEM. Group comparisons were analyzed by an unpaired Student’s *t*-test or one-way ANOVA with Tukey's multiple comparisons test. The Holm procedure was used for multiple comparison adjustment. Linear regression analysis was performed to assess the relationship among parameters. For the logistic regression comparing asthmatic and healthy subjects, an elastic net regression was used with leave-half-out validation for model selection and error estimation. For the logistic regression comparing controlled to uncontrolled asthma, a least squares regression predicting FEV_1_%, stepwise selection was used with the Bayesian Information Criterion as the model selection criterion. All calculations were performed in R (version 3.0.2). The software package GraphPad Prism 6 (GraphPad Software, San Diego, CA) was used for the preparation of graphs. Statistical significance was set at p<0.05.

## Results

### Differences in BAL fluid cellular and cytokine profiles of subjects with asthma vs. healthy controls

**Fig 1:**
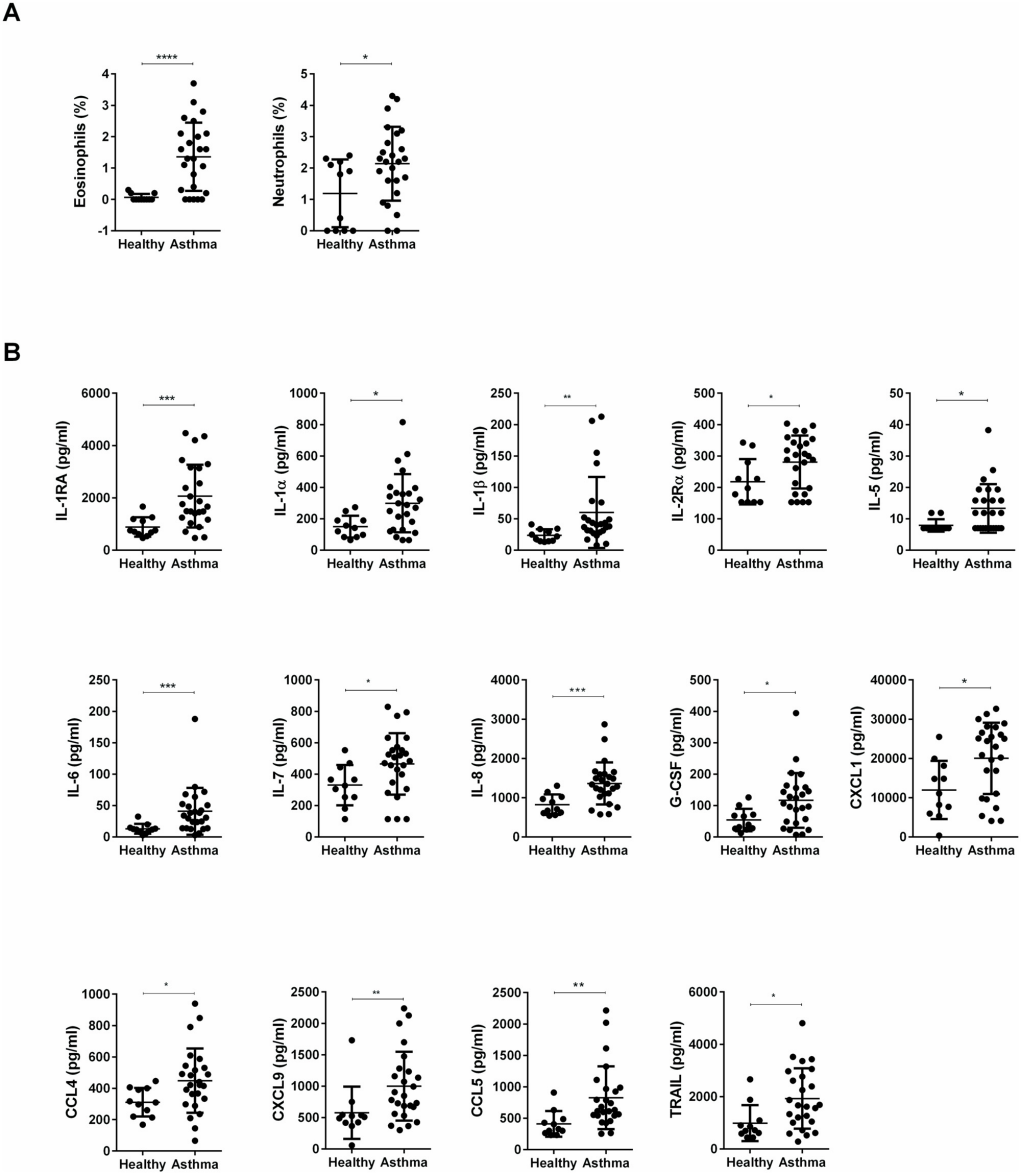
Differences in cell and cytokine levels in BAL fluids in healthy controls vs. asthma. (A) Percentages of eosinophils and neutrophils in the BAL fluids. (B) Concentrations of IL-1RA, IL-1α, IL-1β, IL-2Rα, IL-5, IL-6, IL-7, IL-8, G-CSF, CXCL1, CCL4, CXCL9, CCL5, and TRAIL in the BAL fluids. Data are expressed as means ± SEM. * = *P* < .05, ** = *P* < .01, *** = *P* < .001, **** = *P* < .0001.

Compared to healthy control subjects (n = 11), subjects with asthma (n = 25) had higher % eosinophils (p<0.001) and %neutrophils (p<0.05) in their BAL fluids (Fig 1A). Furthermore, subjects with asthma had 2.3-fold higher IL-1RA (p<0.001), 2.0-fold higher IL-1α (p<0.05), 2.5-fold higher IL-1β (p<0.01), 1.3-fold higher IL-2Rα (p<0.05), 1.7-fold higher IL-5 (p<0.05), 3.2-fold higher IL-6 (p<0.001), 1.4-fold higher IL-7 (p<0.05), 1.7-fold higher IL-8 (p<0.001), 2.2-fold higher G-CSF (p<0.05), 1.7-fold higher CXCL1 (p<0.05), 1.4-fold higher CCL4 (p<0.05), 1.7-fold higher CXCL9 (p<0.01), 2.0-fold higher CCL5 (p<0.01) and 1.9-fold higher TRAIL (p<0.05) concentrations in their BAL fluids (Fig 1B). By contrast, subjects with asthma and healthy controls had similar mean concentrations of such other cytokines as IL-3, IL-9, IL-10, IL-12 (p40), IL-12 (p70), IL-13, IL-15, IL-16, IL-18, IFN-γ, IFN-α2, CXCL10, CCL2, CCL3, PDGF-bb, VEGF, CCL27, HGF, LIF, CCL7, M-CSF, MIF, NGF-β, SCF, SCGF-β, SDF-1α and TNF-β (Table 2). IL-2, IL-4, IL-17, CCL11, FGF, GM-CSF, and TNF-α were not detected in either group. Thus, 14 out of 48 cytokines were higher in subjects with asthma, compared to healthy control subjects.

**Table 2:** Cellular and cytokine profile in BAL fluid without statistical difference between subjects with asthma and healthy controls.

Cellular component and cytokines	Healthy	Asthmatic
IL-2 (pg/ml)	ND	ND
IL-3 (pg/ml)	90.4±103.9	138.2±110.2
IL-4 (pg/ml)	ND	ND
IL-9 (pg/ml)	25.0±9.8	31.5±10.9
IL-10 (pg/ml)	12.8±12.5	14.3±11.9
IL-12 (p40) (pg/ml)	693.5±231.0	811.8±240.4
IL-12 (p70) (pg/ml)	237.1±82.0	233.1±94.4
IL-13 (pg/ml)	12.0±5.2	14.3±6.3
IL-15 (pg/ml)	6.1±4.5	5.8±3.8
IL-16 (pg/ml)	1034.1±935.3	1317.6±749.7
IL-17 (pg/ml)	ND	ND
IL-18 (pg/ml)	282.0±102.9	408.4±262.2
FGF (pg/ml)	ND	ND
CCL11 (pg/ml)	ND	ND
GM-CSF (pg/ml)	ND	ND
IFN-α2 (pg/ml)	105.3±57.1	126.0±47.0
IFN-γ (pg/ml)	73.6±70.2	88.5±89.8
CXCL10 (pg/ml)	13362.5±19043.1	14797.3±10298.6
CCL2 (pg/ml)	349.5±122.5	403.9±132.9
CCL3 (pg/ml)	ND	ND
PDGF-bb (pg/ml)	95.1±94.2	192.5±147.9
TNF-α (pg/ml)	ND	ND
VEGF (pg/ml)	3304.9±1427.3	3358.5±1534.4
CCL27 (pg/ml)	365.0±144.6	416.0±134.5
HGF (pg/ml)	554.5±286.6	748.4±324.3
LIF (pg/ml)	150.3±82.9	168.3±88.0
CCL7 (pg/ml)	522.9±301.4	588.2±243.5
M-CSF (pg/ml)	274.7±103.7	369.1±157.2
MIF (pg/ml)	9627.4±8797.7	14690.1±8929.0
NGF-β (pg/ml)	340.8±87.5	383.7±89.5
SCF (pg/ml)	413.5±222.8	450.1±203.1
SCGF-β (pg/ml)	775.1±546.1	647.5±498.0
SDF-1α (pg/ml)	1118.5±477.5	1427.2±478.7
TNF-β (pg/ml)	582.6±187.4	630.5±208.7

Results expressed in means and range.

ND; Not detected.

### BAL cytokine profile analysis identify only IL-8 levels and % neutrophils as biomarker that distinguish controlled asthma from uncontrolled asthma, and both correlate inversely with FEV_1_

**Fig 2:**
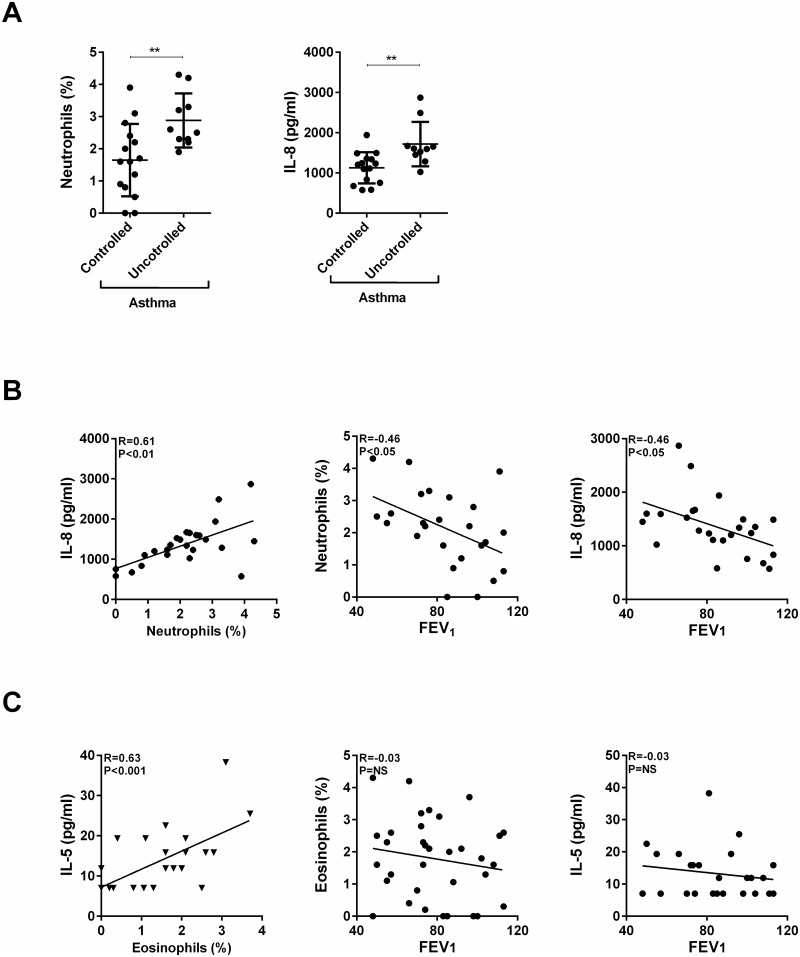
Correlation of FEV1 to eosinophil, neutrophil, IL-5 and IL-8 levels in asthma. (A) Percentages of neutrophils and concentrations of IL-8 in BAL fluids of subjects with controlled asthma and uncontrolled asthma. (B) Correlations of concentrations of IL-8 with the percentages of neutrophils in the BAL fluid from all subjects with asthma (left panel). Correlation of percentages of neutrophils and concentrations of IL-8 in BAL fluid with percent predicted FEV_1_ (middle and right panels, respectively). (C) Correlation of concentrations of IL-5 with the percentages of eosinophil in the BAL fluid from all subjects with asthma (left panel). Correlation of percentages of eosinophils and concentrations of IL-5 in BAL fluid with percent predicted FEV_1_ (middle and right panels, respectively). Data are expressed as means ± SEM. * = *P* < .05, ** = *P* < .01, *** = *P* < .001, **** = P < .0001.

Next, we determined which of these cells and 14 cytokines (Fig 1) elevated in asthma distinguished controlled from uncontrolled asthma. Unexpectedly, there were only two differences between these two groups. Subjects with uncontrolled asthma had a mean 1.7-fold higher percentage of neutrophils in the BAL fluid compared to those with controlled asthma (controlled asthma = 1.6±1.1%, uncontrolled asthma = 2.9±0.8%, p<0.01, Fig 2A). The mean concentration of IL-8 in the BAL fluid from subjects with uncontrolled asthma was 1.5-fold higher than that in subjects with controlled asthma (controlled asthma = 1128±386 pg/ml, uncontrolled asthma = 1716±551 pg/ml, p<0.01, Fig 2A). Furthermore, only IL-8 concentrations in all subjects with asthma (controlled and uncontrolled) significantly correlated with the percentages of neutrophils in the BAL fluid (R = 0.61, p<0.01, Fig 2B). In addition, the percentages of neutrophils and the concentrations of IL-8 in the BAL fluid were both inversely correlated with the % predicted FEV_1_ (R = -0.46, p<0.05 for both neutrophil% and IL-8 levels, Fig 2B). Even though BAL eosinophil % in all subjects with asthma correlated with BAL fluid IL-5 levels (Fig 2C), neither eosinophil % nor IL-5 levels correlated with % predicted FEV_1_ (Fig 2C). Some cytokines elevated in subjects with asthma significantly correlated with the level of IL-8 in BAL fluids: IL1-RA (R = 0.59, p<0.01), IL-1α (R = 0.40, p<0.05), IL-6 (R = 0.68, p<0.001), IL-7 (R = 0.47, p<0.05), G-CSF (R = 0.74, p<0.0001), CCL4 (R = 0.45, p<0.05), CXCL1 (R = 0.64, p<0.01), and CXCL9 (R = 0.48, p<0.05). However, these cytokines did not correlate with the % neutrophils or % predicted FEV_1_ in BAL fluids.

Next we statistically examined whether inhaled corticosteroid (ICS) could have contributed to some of the observations in the present study by separating all subjects with asthma into those that received ICS vs. those that did not. Subjects with asthma that were being treated with ICS had higher % neutrophils (p<0.05), higher IL-8 levels (p<0.05) and lower % predicted FEV_1_ (p<0.0001). However, the dose of ICS did not correlate the level of % neutrophils and IL-8 levels in BAL fluids (data not shown).

### Eosinophil-high and neutrophil-high asthma have different cytokine profiles and FEV_1_

**Fig 3:**
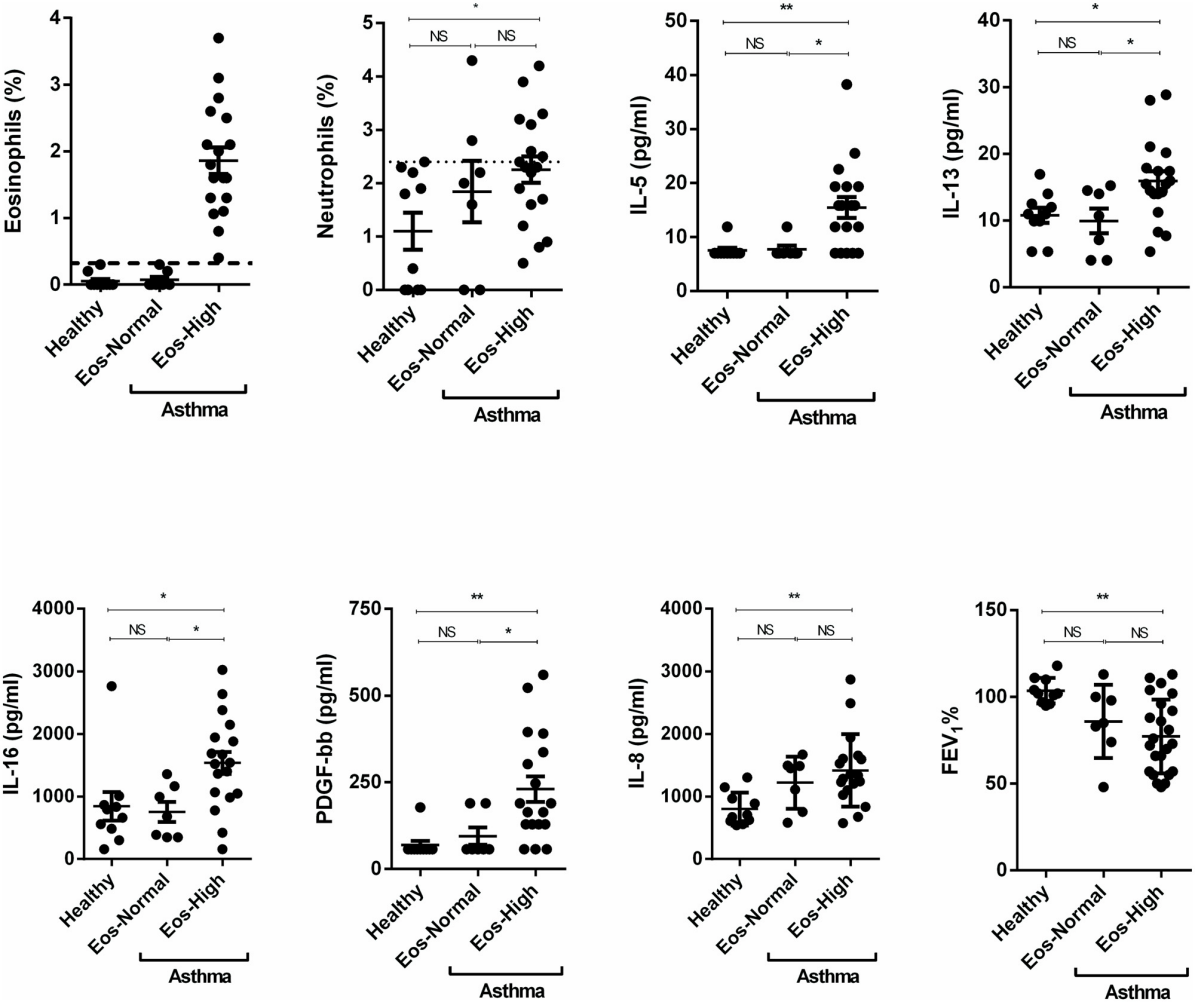
Cell and cytokine profile of eosinophil-high (Eos-High) asthma and eosinophil-normal (Eos-Normal) asthma. The upper limit of percent of eosinophils in the BAL fluid of healthy subjects was 0.3%. We separated all subjects with asthma into either eosinophil-high (eosinophils > 0.3%) and eosinophil-normal (eosinophils≤0.3%) groups. Compared to Eos-Normal asthma, Eos-High asthma had higher levels of IL-5 (p<0.05), IL-13 (p<0.05), IL-16 (p<0.05), and PDGF-bb (p<0.05), but same % neutrophils, IL-8, and FEV_1_. Data are expressed as means ± SEM. *P < .05, **P < .01.

**Fig 4:**
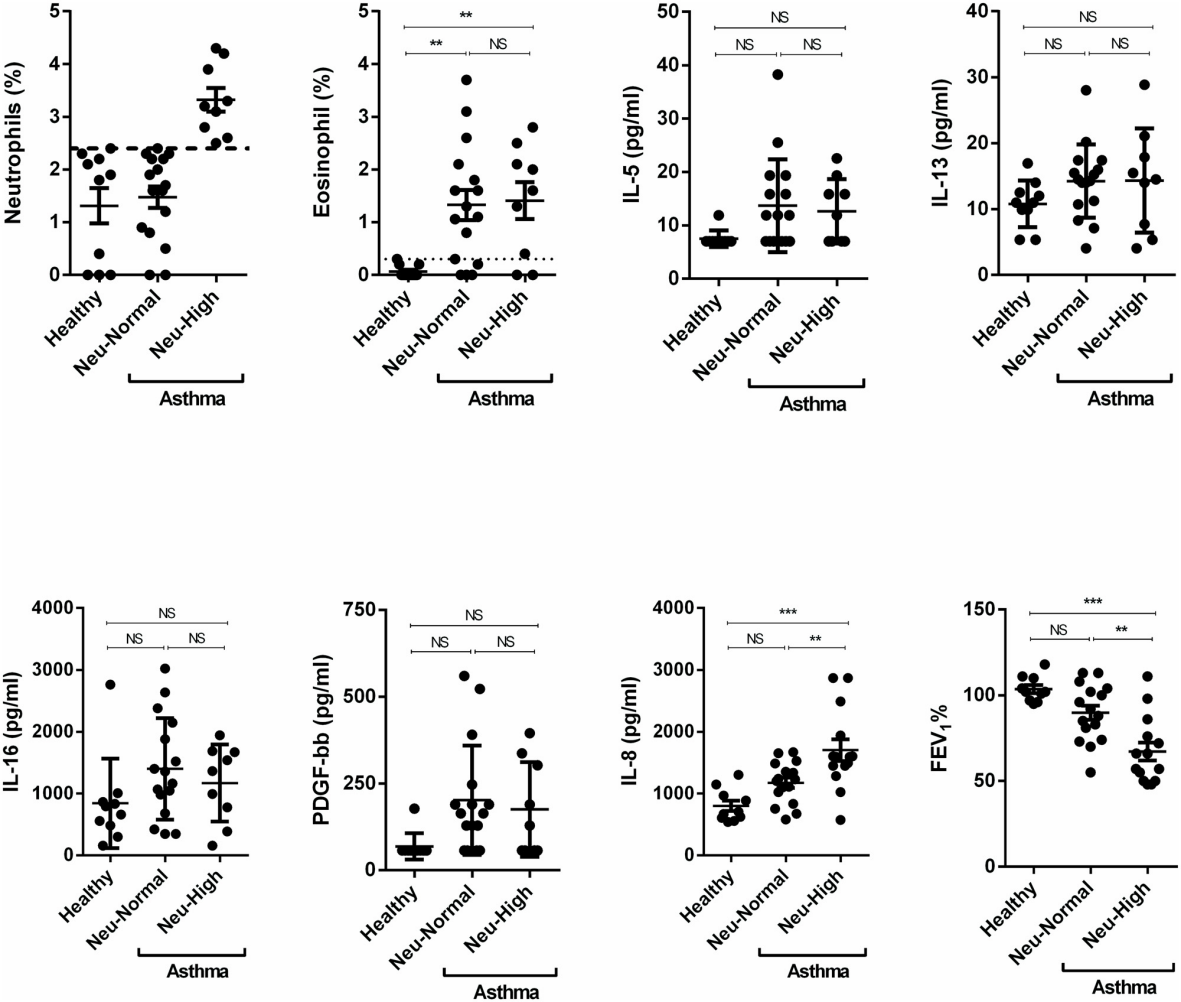
Cell and cytokine profile of neutrophil-high (Neu-High) asthma and neutrophil-normal (Neu-Normal) asthma. The upper limit of percent of neutrophils in the BAL fluid of healthy subjects was 2.4%. We separated all subjects with asthma into neutrophil-high (neutrophils% > 2.4%), and neutrophil-normal (neutrophil≤2.4%) groups. Compared to Neu-Normal asthma, Neu-High asthma had higher IL-8 levels (p<0.01) and lower % predicted FEV_1_ (p<0.01), but similar levels of eosinophil %, IL-5, IL-13, IL-16, and PDGF-bb. Data are expressed as means ± SEM. *P < .05, **P < .01.

Building on the unexpected observation that % neutrophil but not % eosinophils correlated inversely with % predicted FEV_1_ in asthma, we examined whether grouping asthma subjects on the basis of BAL eosinophil % or neutrophil % could identify specific cytokine profiles. In our study, the upper limit of percent of eosinophils and neutrophils in the BAL fluid of healthy subjects was 0.3% and 2.4%, respectively (Figs 3 and 4). For the purpose of this study, we separated all subjects with asthma into either eosinophil-high (eosinophils > 0.3%, Eos-High) and eosinophil-normal (eosinophils≤0.3%, Eos-Normal) groups (Fig 3), or neutrophil-high (neutrophils% > 2.4%, Neu-High), and neutrophil-normal (neutrophil≤2.4%, Neu-Normal) groups (Fig 4). Compared to Eos-Normal asthma, Eos-High asthma had higher levels of IL-5 (p<0.05), IL-13 (p<0.05), IL-16 (p<0.05), and PDGF-bb (p<0.05), but same % neutrophils, IL-8, other cytokines (data not shown), and FEV_1_ (Fig 3). By contrast, compared to Neu-Normal asthma, Neu-High asthma had higher IL-8 levels (p<0.01) and lower % predicted FEV_1_ (p<0.01), but similar levels of eosinophil %, IL-5, IL-13, IL-16, and PDGF-bb (Fig 4) and other cytokines and chemokines (data not shown). These results also indicate an association of Neu-High asthma with IL-8 and % FEV_1_.

### Multiple regression analysis models

The estimated predictive equation for the presence of asthma using logistic regression was: Logit (Present (asthma)) = -3.85 + 0.0033 (IL-8) + 2.77 (% eosinophils) (p = 0.05 and 0.09, respectively). The accuracy of this model was 84%, with 89% sensitivity and 75% specificity. The predictive equation for FEV_1_% predicted in asthma was 103–0.023 (IL-8) + 0.040 (IL-1α). The R^2^ for this model was 0.34 (p = 0.0037 and 0.06, respectively). Atopy had no significant effect.

## Discussion

Prior studies have mostly measured candidate cytokines, and reported increased levels of IL-8 and neutrophils in the sputum in severe asthma [7]. Our study of the BAL fluid provides this specific information by demonstrating that IL-8 is the only cytokine among 48 measured that is significantly elevated in uncontrolled asthma. The higher BAL fluid IL-8 levels in uncontrolled asthma seen in our study could reflect persistent stimulation of IL-8 secretion by chronic stimulation of the nuclear factor-κB signaling pathway following exposure to environemantal factor [19], or intrinsic differences in the ability of uncontrolled asthma patients’ airway epithelium to produce high amounts of IL-8 [20]. In addition to its ability to stimulate neutrophil recruitment, IL-8 may contribute to the pathogenesis of severe asthma by directly facilitating airway remodeling by increasing bronchial smooth muscle cell migration and proliferation [21], inducing airway hyperresponsiveness (AHR) [22], and stimulating epithelial-mesenchymal transition (EMT) [23] in the airways.

In our study, neutrophil-high asthma had lower FEV_1_, and the neutrophil percentage in asthma was inversely correlated with FEV_1_ and directly correlated with IL-8 levels. The mechanistic contribution of neutrophils to asthma severity is not well understood, and our study was not designed to address this issue. A variety of factors produced by neutrophils could theoretically contribute to the pathogenesis of severe asthma. Depletion of neutrophils in a mouse model of allergic asthma has been reported to reduce AHR and airway remodeling [24]. Matrix metalloproteinase 9 (MMP-9) from neutrophils has been shown to be associated with asthma severity [24]. Neutrophil elastase can induce AHR [25], and promote the EMT [26]. After interacting with allergens, neutrophils release α-defensins [27], which can stimulate IL-8 secretion from human bronchial epithelial cells [28]. Neutrophils from subjects with asthma produce higher TGF-β1 [29], a strong inducer of the EMT. Neutrophils are a major source of reactive oxygen species (ROS) generated by gp91phox NADPH oxidase [30], and promote allergic airway inflammation [31].

In our study, 12% of the subjects with controlled asthma and all subjects with uncontrolled asthma used ICS. Because steroids can inhibit apoptosis of neutrophils [32] and suppress eosinophil survival [33], use of ICS could have impacted the results of our study by skewing cell counts to higher neutrophilia in uncontrolled asthma. However, in our study the dose of ICS did not correlated the level of neutrophils in BAL fluids, suggesting that this is most likely not the explanation for higher %neutrophil. As in our study, others have also reported elevated neutrophils in severe asthma, independent of steroids. For example, the European Network for Understanding Mechanisms of Severe Asthma study also reported more neutrophils in the sputum from subjects with severe asthma, independent of corticosteroid use [34]. Likewise, use of inhaled corticosteroids did not impact BAL fluid IL-8 levels in a study of the molecular phenotyping of severe asthma [16]. Further studies are needed to clarify the effect of ICS on neutrophils and eosinophils in the airways [35].

Consistent with prior studies [11, 12, 36–43], our results also demonstrate that subjects with asthma have higher concentrations of IL-5 and the numbers of eosinophils in BAL fluid compared to control subjects. This is not surprising because eosinophilic inflammation is a significant feature of the pathology of asthma [9–12, 36, 37, 44]. In our study, eosinophils and IL-5 did not correlate with percent predicted FEV_1_. The lack of association between eosinophils and FEV_1_ in asthma is surprising because eosinophils have been shown to contribute to AHR in murine, guinea pig and mammal studies [45–48]. However, several human studies have shown that eosinophils do not correlate with AHR or airflow obstruction [49–51].

It is somewhat surprising some Th2 cytokines and chemokines, especially IL-4, IL-13, and CCL11 were not elevated in the present study, even though prior reports indicated the increase of these cytokines and chemokines [38–43, 52–58]. Two studies performed in the 1990s reported elevated IL-4 levels in concentrated BAL fluids in asthma [40, 41]. Since concentrating BAL fluid may induce a processing artifact, more recent studies have been performed on unconcentrated BAL fluids [15, 59]. Like our study that was also performed on unconcentrated BAL fluids using multiplex beads, these studies reported that IL-4 and IL-13 were undetectable in unconcentrated BAL fluids in asthma [15, 59]. Like our study, a previous study reported that there is no elevation of TNFα or GM-CSF in BAL fluids from the subjects of asthma [15]. Prior studies have reported an increase in CCL11 levels in BAL fluids in subjects with asthma after allergen challenge [56], and CCL11positive cells or CCL11 mRNA expression in bronchial biopsy specimens in asthma [57, 58]. However, other studies that were similar to ours, and sampled the BAL compartment in asthma without allergen challenge, also failed to detect CCL11 [60], or detected CCL11 at a level that would be too low (7–41 pg/ml) to be detectable by our kit (lower limit of detection 325 pg/ml) [15, 59].

We unexpectedly did not detect IL-17in our study. A recent study reported that IL-17 is present in BAL fluids from the subjects with asthma at mean levels of about 60 pg/ml (25–150 pg/ml) [61]. Since the lower limit of detection level of IL-17 in our study 48 pg/ml, this could account for failure to detect IL-17 in our study.

Recent studies have suggested that asthma is a heterogeneous disease complex that should be classified into distinct endotypes based on their cytokine profiles [15, 16, 62–65]. In the present study we also show there are quantitative differences in cytokine pattern between neutrophil-high asthma and eosinophil-high asthma. However, our data suggest that uncontrolled and controlled asthma have a fairly uniform cytokine profile and may have a common pathogenesis instead of being a collection of fundamentally distinct diseases. Our observations question the importance cytokine-based endotypes classification of asthma in predicting asthma severity.

The specific association of only IL-8 in 48 cytokines quantified in BAL fluids with neutrophil-high and uncontrolled asthma in the present study provides specificity to earlier candidate-cytokine studies reporting elevated IL-8 and neutrophils in severe asthma [7–10]. Together, these studies indicate that the mechanistic role of IL-8 and recruited neutrophils should be carefully evaluated in uncontrolled asthma. CXCR2 is one of the receptors for IL-8 [66]. A recent study demonstrated that CXCR2 inhibitor reduced sputum neutrophilia and asthma exacerbations, and improved Asthma Control Questionnaire (ACQ) score in patients with severe asthma [67]. If the results of our study are confirmed in mechanistic and large-scale BAL fluid studies, inhibition of neutrophil recruitment by CXCR2 inhibitors and others agents should be explored as alternate therapeutic strategies in uncontrolled asthma with elevated neutrophils.

## References

[1] Braman SS. The global burden of asthma. Chest. 2006;130(1 Suppl):4S–12S. Epub 2006/07/15. doi: 130/1_suppl/4S [pii] 10.1378/chest.130.1_suppl.4S . 16840363

[2] Godard P, Chanez P, Siraudin L, Nicoloyannis N, Duru G. Costs of asthma are correlated with severity: a 1-yr prospective study. Eur Respir J. 2002;19(1):61–7. Epub 2002/02/15. . 11843329 10.1183/09031936.02.00232001

[3] Anagnostou K, Harrison B, Iles R, Nasser S. Risk factors for childhood asthma deaths from the UK Eastern Region Confidential Enquiry 2001–2006. Prim Care Respir J. 2012;21(1):71–7. Epub 2012/01/06. 10.4104/pcrj.2011.00097 pcrj-2011-05-0059-R2 [pii]. 10.4104/pcrj.2011.00097 22218820 PMC6547890

[4] Moore WC, Bleecker ER, Curran-Everett D, Erzurum SC, Ameredes BT, Bacharier L, et al Characterization of the severe asthma phenotype by the National Heart, Lung, and Blood Institute's Severe Asthma Research Program. J Allergy Clin Immunol. 2007;119(2):405–13. Epub 2007/02/13. doi: S0091-6749(06)03030-2 [pii] 10.1016/j.jaci.2006.11.639 17291857 PMC2837934

[5] Wenzel SE, Schwartz LB, Langmack EL, Halliday JL, Trudeau JB, Gibbs RL, et al Evidence that severe asthma can be divided pathologically into two inflammatory subtypes with distinct physiologic and clinical characteristics. Am J Respir Crit Care Med. 1999;160(3):1001–8. Epub 1999/09/03. 10.1164/ajrccm.160.3.9812110 . 10471631

[6] Sur S, Crotty TB, Kephart GM, Hyma BA, Colby TV, Reed CE, et al Sudden-onset fatal asthma. A distinct entity with few eosinophils and relatively more neutrophils in the airway submucosa? Am Rev Respir Dis. 1993;148(3):713–9. Epub 1993/09/01. . 8368644 10.1164/ajrccm/148.3.713

[7] Jatakanon A, Uasuf C, Maziak W, Lim S, Chung KF, Barnes PJ. Neutrophilic inflammation in severe persistent asthma. Am J Respir Crit Care Med. 1999;160(5 Pt 1):1532–9. Epub 1999/11/11. . 10556116 10.1164/ajrccm.160.5.9806170

[8] Moore WC, Hastie AT, Li X, Li H, Busse WW, Jarjour NN, et al Sputum neutrophil counts are associated with more severe asthma phenotypes using cluster analysis. J Allergy Clin Immunol. 2013. Epub 2013/12/18. doi: S0091-6749(13)01563-7 [pii] 10.1016/j.jaci.2013.10.011 . 24332216 PMC4040309

[9] Ordonez CL, Shaughnessy TE, Matthay MA, Fahy JV. Increased neutrophil numbers and IL-8 levels in airway secretions in acute severe asthma: Clinical and biologic significance. Am J Respir Crit Care Med. 2000;161(4 Pt 1):1185–90. Epub 2000/04/14. . 10764310 10.1164/ajrccm.161.4.9812061

[10] Lamblin C, Gosset P, Tillie-Leblond I, Saulnier F, Marquette CH, Wallaert B, et al Bronchial neutrophilia in patients with noninfectious status asthmaticus. Am J Respir Crit Care Med. 1998;157(2):394–402. Epub 1998/02/26. 10.1164/ajrccm.157.2.97-02099 . 9476849

[11] Sur S, Gleich GJ, Swanson MC, Bartemes KR, Broide DH. Eosinophilic inflammation is associated with elevation of interleukin-5 in the airways of patients with spontaneous symptomatic asthma. J Allergy Clin Immunol. 1995;96(5 Pt 1):661–8. Epub 1995/11/01. doi: S0091674995001904 [pii]. . 7499683 10.1016/s0091-6749(95)70265-2

[12] Sur S, Kita H, Gleich GJ, Chenier TC, Hunt LW. Eosinophil recruitment is associated with IL-5, but not with RANTES, twenty-four hours after allergen challenge. J Allergy Clin Immunol. 1996;97(6):1272–8. Epub 1996/06/01. doi: S0091-6749(96)70195-1 [pii]. . 8648023 10.1016/s0091-6749(96)70195-1

[13] Arron JR, Choy DF, Laviolette M, Kelsen SG, Hatab A, Leigh R, et al Disconnect between sputum neutrophils and other measures of airway inflammation in asthma. Eur Respir J. 2013. Epub 2013/11/02. doi: 09031936.00117013 [pii] 10.1183/09031936.00117013 . 24176993

[14] Wenzel SE. Asthma phenotypes: the evolution from clinical to molecular approaches. Nat Med. 2012;18(5):716–25. Epub 2012/05/09. 10.1038/nm.2678 nm.2678 [pii]. 10.1038/nm.2678 22561835

[15] Fitzpatrick AM, Higgins M, Holguin F, Brown LA, Teague WG. The molecular phenotype of severe asthma in children. J Allergy Clin Immunol. 2010;125(4):851–7 e18 Epub 2010/04/08. 10.1016/j.jaci.2010.01.048 S0091-6749(10)00181-8 [pii]. 10.1016/j.jaci.2010.01.048 20371397 PMC2851636

[16] Brasier AR, Victor S, Boetticher G, Ju H, Lee C, Bleecker ER, et al Molecular phenotyping of severe asthma using pattern recognition of bronchoalveolar lavage-derived cytokines. J Allergy Clin Immunol. 2008;121(1):30–7 e6. Epub 2008/01/22. doi: S0091-6749(07)01960-4 [pii] 10.1016/j.jaci.2007.10.015 18206505 PMC3019566

[17] Chung KF, Wenzel SE, Brozek JL, Bush A, Castro M, Sterk PJ, et al International ERS/ATS guidelines on definition, evaluation and treatment of severe asthma. Eur Respir J. 2014;43(2):343–73. Epub 2013/12/18. 10.1183/09031936.00202013 09031936.00202013 [pii]. 10.1183/09031936.00202013 24337046

[18] Ying S, O'Connor B, Ratoff J, Meng Q, Fang C, Cousins D, et al Expression and cellular provenance of thymic stromal lymphopoietin and chemokines in patients with severe asthma and chronic obstructive pulmonary disease. J Immunol. 2008;181(4):2790–8. Epub 2008/08/08. doi: 181/4/2790 [pii]. . 18684970 10.4049/jimmunol.181.4.2790

[19] Gagliardo R, Chanez P, Mathieu M, Bruno A, Costanzo G, Gougat C, et al Persistent activation of nuclear factor-kappaB signaling pathway in severe uncontrolled asthma. Am J Respir Crit Care Med. 2003;168(10):1190–8. Epub 2003/08/02. 10.1164/rccm.200205-479OC 200205-479OC [pii]. . 12893643 10.1164/rccm.200205-479OC

[20] Gras D, Bourdin A, Vachier I, de Senneville L, Bonnans C, Chanez P. An ex vivo model of severe asthma using reconstituted human bronchial epithelium. J Allergy Clin Immunol. 2012;129(5):1259–66 e1 Epub 2012/03/14. 10.1016/j.jaci.2012.01.073 S0091-6749(12)00261-8 [pii]. 10.1016/j.jaci.2012.01.073 22409990

[21] Kuo PL, Hsu YL, Huang MS, Chiang SL, Ko YC. Bronchial epithelium-derived IL-8 and RANTES increased bronchial smooth muscle cell migration and proliferation by Kruppel-like factor 5 in areca nut-mediated airway remodeling. Toxicol Sci. 2011;121(1):177–90. Epub 2011/02/08. 10.1093/toxsci/kfr030 kfr030 [pii]. 10.1093/toxsci/kfr030 21297082

[22] Xiu Q, Fujimura M, Nomura M, Saito M, Matsuda T, Akao N, et al Bronchial hyperresponsiveness and airway neutrophil accumulation induced by interleukin-8 and the effect of the thromboxane A2 antagonist S-1452 in guinea-pigs. Clin Exp Allergy. 1995;25(1):51–9. Epub 1995/01/01. . 7728625 10.1111/j.1365-2222.1995.tb01002.x

[23] Fernando RI, Castillo MD, Litzinger M, Hamilton DH, Palena C. IL-8 signaling plays a critical role in the epithelial-mesenchymal transition of human carcinoma cells. Cancer Res. 2011;71(15):5296–306. Epub 2011/06/10. 10.1158/0008-5472.CAN-11-0156 0008-5472.CAN-11-0156 [pii]. 10.1158/0008-5472.CAN-11-0156 21653678 PMC3148346

[24] Park SJ, Wiekowski MT, Lira SA, Mehrad B. Neutrophils regulate airway responses in a model of fungal allergic airways disease. J Immunol. 2006;176(4):2538–45. Epub 2006/02/04. doi: 176/4/2538 [pii]. . 16456015 10.4049/jimmunol.176.4.2538

[25] Suzuki T, Wang W, Lin JT, Shirato K, Mitsuhashi H, Inoue H. Aerosolized human neutrophil elastase induces airway constriction and hyperresponsiveness with protection by intravenous pretreatment with half-length secretory leukoprotease inhibitor. Am J Respir Crit Care Med. 1996;153(4 Pt 1):1405–11. Epub 1996/04/01. 10.1164/ajrccm.153.4.8616573 . 8616573

[26] Grosse-Steffen T, Giese T, Giese N, Longerich T, Schirmacher P, Hansch GM, et al Epithelial-to-mesenchymal transition in pancreatic ductal adenocarcinoma and pancreatic tumor cell lines: the role of neutrophils and neutrophil-derived elastase. Clin Dev Immunol. 2012;2012:720768 Epub 2012/12/12. 10.1155/2012/720768 23227088 PMC3514849

[27] Vega A, Ventura I, Chamorro C, Aroca R, Orovigt A, Gomez E, et al Neutrophil defensins: their possible role in allergic asthma. J Investig Allergol Clin Immunol. 2011;21(1):38–43. Epub 2011/03/05. . 21370722

[28] Sakamoto N, Mukae H, Fujii T, Ishii H, Yoshioka S, Kakugawa T, et al Differential effects of alpha- and beta-defensin on cytokine production by cultured human bronchial epithelial cells. Am J Physiol Lung Cell Mol Physiol. 2005;288(3):L508–13. Epub 2004/11/24. doi: 00076.2004 [pii] 10.1152/ajplung.00076.2004 . 15557089

[29] Chu HW, Trudeau JB, Balzar S, Wenzel SE. Peripheral blood and airway tissue expression of transforming growth factor beta by neutrophils in asthmatic subjects and normal control subjects. J Allergy Clin Immunol. 2000;106(6):1115–23. Epub 2000/12/12. doi: S0091-6749(00)54752-6 [pii] 10.1067/mai.2000.110556 . 11112895

[30] Hampton MB, Kettle AJ, Winterbourn CC. Inside the neutrophil phagosome: oxidants, myeloperoxidase, and bacterial killing. Blood. 1998;92(9):3007–17. Epub 1998/10/27. . 9787133

[31] Sevin CM, Newcomb DC, Toki S, Han W, Sherrill TP, Boswell MG, et al Deficiency of gp91phox inhibits allergic airway inflammation. Am J Respir Cell Mol Biol. 2013;49(3):396–402. Epub 2013/04/18. 10.1165/rcmb.2012-0442OC 23590311 PMC3824054

[32] Cox G. Glucocorticoid treatment inhibits apoptosis in human neutrophils. Separation of survival and activation outcomes. J Immunol. 1995;154(9):4719–25. Epub 1995/05/01. . 7722324

[33] Cox G, Ohtoshi T, Vancheri C, Denburg JA, Dolovich J, Gauldie J, et al Promotion of eosinophil survival by human bronchial epithelial cells and its modulation by steroids. Am J Respir Cell Mol Biol. 1991;4(6):525–31. Epub 1991/06/01. 10.1165/ajrcmb/4.6.525 . 2054193

[34] The ENFUMOSA cross-sectional European multicentre study of the clinical phenotype of chronic severe asthma. European Network for Understanding Mechanisms of Severe Asthma. Eur Respir J. 2003;22(3):470–7. Epub 2003/10/01. . 14516137 10.1183/09031936.03.00261903

[35] Green RH, Brightling CE, Woltmann G, Parker D, Wardlaw AJ, Pavord ID. Analysis of induced sputum in adults with asthma: identification of subgroup with isolated sputum neutrophilia and poor response to inhaled corticosteroids. Thorax. 2002;57(10):875–9. Epub 2002/09/27. 12324674 10.1136/thorax.57.10.875 PMC1746199

[36] Virchow JC Jr., Walker C, Hafner D, Kortsik C, Werner P, Matthys H, et al T cells and cytokines in bronchoalveolar lavage fluid after segmental allergen provocation in atopic asthma. Am J Respir Crit Care Med. 1995;151(4):960–8. Epub 1995/04/01. 10.1164/ajrccm/151.4.960 . 7697273

[37] Tillie-Leblond I, Hammad H, Desurmont S, Pugin J, Wallaert B, Tonnel AB, et al CC chemokines and interleukin-5 in bronchial lavage fluid from patients with status asthmaticus. Potential implication in eosinophil recruitment. Am J Respir Crit Care Med. 2000;162(2 Pt 1):586–92. Epub 2000/08/10. 10.1164/ajrccm.162.2.9907014 . 10934091

[38] Ying S, Humbert M, Barkans J, Corrigan CJ, Pfister R, Menz G, et al Expression of IL-4 and IL-5 mRNA and protein product by CD4+ and CD8+ T cells, eosinophils, and mast cells in bronchial biopsies obtained from atopic and nonatopic (intrinsic) asthmatics. J Immunol. 1997;158(7):3539–44. Epub 1997/04/01. . 9120316

[39] Humbert M, Durham SR, Ying S, Kimmitt P, Barkans J, Assoufi B, et al IL-4 and IL-5 mRNA and protein in bronchial biopsies from patients with atopic and nonatopic asthma: evidence against "intrinsic" asthma being a distinct immunopathologic entity. Am J Respir Crit Care Med. 1996;154(5):1497–504. Epub 1996/11/01. 10.1164/ajrccm.154.5.8912771 . 8912771

[40] Walker C, Bauer W, Braun RK, Menz G, Braun P, Schwarz F, et al Activated T cells and cytokines in bronchoalveolar lavages from patients with various lung diseases associated with eosinophilia. Am J Respir Crit Care Med. 1994;150(4):1038–48. Epub 1994/10/01. . 7921434 10.1164/ajrccm.150.4.7921434

[41] Walker C, Bode E, Boer L, Hansel TT, Blaser K, Virchow JC Jr. Allergic and nonallergic asthmatics have distinct patterns of T-cell activation and cytokine production in peripheral blood and bronchoalveolar lavage. Am Rev Respir Dis. 1992;146(1):109–15. Epub 1992/07/11. 10.1164/ajrccm/146.1.109 . 1626792

[42] Leung DY, Martin RJ, Szefler SJ, Sher ER, Ying S, Kay AB, et al Dysregulation of interleukin 4, interleukin 5, and interferon gamma gene expression in steroid-resistant asthma. J Exp Med. 1995;181(1):33–40. Epub 1995/01/01. . 7807013 10.1084/jem.181.1.33 PMC2191836

[43] Robinson DS, Hamid Q, Ying S, Tsicopoulos A, Barkans J, Bentley AM, et al Predominant TH2-like bronchoalveolar T-lymphocyte population in atopic asthma. N Engl J Med. 1992;326(5):298–304. Epub 1992/01/30. 10.1056/NEJM199201303260504 . 1530827

[44] Wardlaw AJ, Dunnette S, Gleich GJ, Collins JV, Kay AB. Eosinophils and mast cells in bronchoalveolar lavage in subjects with mild asthma. Relationship to bronchial hyperreactivity. Am Rev Respir Dis. 1988;137(1):62–9. Epub 1988/01/01. 10.1164/ajrccm/137.1.62 . 2447813

[45] Lee JJ, Dimina D, Macias MP, Ochkur SI, McGarry MP, O'Neill KR, et al Defining a link with asthma in mice congenitally deficient in eosinophils. Science. 2004;305(5691):1773–6. Epub 2004/09/18. 10.1126/science.1099472 305/5691/1773 [pii]. . 15375267 10.1126/science.1099472

[46] Justice JP, Borchers MT, Crosby JR, Hines EM, Shen HH, Ochkur SI, et al Ablation of eosinophils leads to a reduction of allergen-induced pulmonary pathology. Am J Physiol Lung Cell Mol Physiol. 2003;284(1):L169–78. Epub 2002/10/22. 10.1152/ajplung.00260.2002 00260.2002 [pii]. . 12388345 10.1152/ajplung.00260.2002

[47] Verbout NG, Jacoby DB, Gleich GJ, Fryer AD. Atropine-enhanced, antigen challenge-induced airway hyperreactivity in guinea pigs is mediated by eosinophils and nerve growth factor. Am J Physiol Lung Cell Mol Physiol. 2009;297(2):L228–37. Epub 2009/05/19. doi: 90540.2008 [pii] 10.1152/ajplung.90540.2008 19447892 PMC2742791

[48] Gundel RH, Letts LG, Gleich GJ. Human eosinophil major basic protein induces airway constriction and airway hyperresponsiveness in primates. J Clin Invest. 1991;87(4):1470–3. Epub 1991/04/01. 10.1172/JCI115155 2010556 PMC295201

[49] Brightling CE, Symon FA, Birring SS, Bradding P, Wardlaw AJ, Pavord ID. Comparison of airway immunopathology of eosinophilic bronchitis and asthma. Thorax. 2003;58(6):528–32. Epub 2003/05/31. 12775868 10.1136/thorax.58.6.528 PMC1746707

[50] Flood-Page PT, Menzies-Gow AN, Kay AB, Robinson DS. Eosinophil's role remains uncertain as anti-interleukin-5 only partially depletes numbers in asthmatic airway. Am J Respir Crit Care Med. 2003;167(2):199–204. Epub 2002/10/31. 10.1164/rccm.200208-789OC 200208-789OC [pii]. . 12406833 10.1164/rccm.200208-789OC

[51] Leckie MJ, ten Brinke A, Khan J, Diamant Z, O'Connor BJ, Walls CM, et al Effects of an interleukin-5 blocking monoclonal antibody on eosinophils, airway hyper-responsiveness, and the late asthmatic response. Lancet. 2000;356(9248):2144–8. Epub 2001/02/24. doi: S0140673600034966 [pii]. . 11191542 10.1016/s0140-6736(00)03496-6

[52] Huang SK, Xiao HQ, Kleine-Tebbe J, Paciotti G, Marsh DG, Lichtenstein LM, et al IL-13 expression at the sites of allergen challenge in patients with asthma. J Immunol. 1995;155(5):2688–94. Epub 1995/09/01. . 7650396

[53] Kotsimbos TC, Ernst P, Hamid QA. Interleukin-13 and interleukin-4 are coexpressed in atopic asthma. Proc Assoc Am Physicians. 1996;108(5):368–73. Epub 1996/09/01. . 8902881

[54] Kroegel C, Julius P, Matthys H, Virchow JC Jr., Luttmann W. Endobronchial secretion of interleukin-13 following local allergen challenge in atopic asthma: relationship to interleukin-4 and eosinophil counts. Eur Respir J. 1996;9(5):899–904. Epub 1996/05/01. . 8793449 10.1183/09031936.96.09050899

[55] Moller GM, de Jong TA, van der Kwast TH, Overbeek SE, Wierenga-Wolf AF, Thepen T, et al Immunolocalization of interleukin-4 in eosinophils in the bronchial mucosa of atopic asthmatics. Am J Respir Cell Mol Biol. 1996;14(5):439–43. Epub 1996/05/01. 10.1165/ajrcmb.14.5.8624248 . 8624248

[56] Lilly CM, Nakamura H, Belostotsky OI, Haley KJ, Garcia-Zepeda EA, Luster AD, et al Eotaxin expression after segmental allergen challenge in subjects with atopic asthma. Am J Respir Crit Care Med. 2001;163(7):1669–75. Epub 2001/06/13. 10.1164/ajrccm.163.7.9812044 . 11401892

[57] Lamkhioued B, Renzi PM, Abi-Younes S, Garcia-Zepada EA, Allakhverdi Z, Ghaffar O, et al Increased expression of eotaxin in bronchoalveolar lavage and airways of asthmatics contributes to the chemotaxis of eosinophils to the site of inflammation. J Immunol. 1997;159(9):4593–601. Epub 1997/10/31. . 9379061

[58] Ying S, Robinson DS, Meng Q, Rottman J, Kennedy R, Ringler DJ, et al Enhanced expression of eotaxin and CCR3 mRNA and protein in atopic asthma. Association with airway hyperresponsiveness and predominant co-localization of eotaxin mRNA to bronchial epithelial and endothelial cells. Eur J Immunol. 1997;27(12):3507–16. Epub 1998/02/17. 10.1002/eji.1830271252 . 9464841

[59] Bossley CJ, Fleming L, Gupta A, Regamey N, Frith J, Oates T, et al Pediatric severe asthma is characterized by eosinophilia and remodeling without T(H)2 cytokines. J Allergy Clin Immunol. 2012;129(4):974–82 e13 Epub 2012/03/06. 10.1016/j.jaci.2012.01.059 S0091-6749(12)00187-X [pii]. 10.1016/j.jaci.2012.01.059 22385633 PMC3381727

[60] Woodman L, Sutcliffe A, Kaur D, Berry M, Bradding P, Pavord ID, et al Chemokine concentrations and mast cell chemotactic activity in BAL fluid in patients with eosinophilic bronchitis and asthma, and in normal control subjects. Chest. 2006;130(2):371–8. Epub 2006/08/11. doi: 130/2/371 [pii] 10.1378/chest.130.2.371 . 16899834

[61] Irvin C, Zafar I, Good J, Rollins D, Christianson C, Gorska MM, et al Increased frequency of dual-positive TH2/TH17 cells in bronchoalveolar lavage fluid characterizes a population of patients with severe asthma. J Allergy Clin Immunol. 2014;134(5):1175–86 e7 Epub 2014/07/22. 10.1016/j.jaci.2014.05.038 S0091-6749(14)00799-4 [pii]. 10.1016/j.jaci.2014.05.038 25042748 PMC4254017

[62] Anderson GP. Endotyping asthma: new insights into key pathogenic mechanisms in a complex, heterogeneous disease. Lancet. 2008;372(9643):1107–19. Epub 2008/09/23. doi: S0140-6736(08)61452-X [pii] 10.1016/S0140-6736(08)61452-X . 18805339

[63] Moore WC, Meyers DA, Wenzel SE, Teague WG, Li H, Li X, et al Identification of asthma phenotypes using cluster analysis in the Severe Asthma Research Program. Am J Respir Crit Care Med. 2010;181(4):315–23. Epub 2009/11/07. 10.1164/rccm.200906-0896OC 200906-0896OC [pii]. 10.1164/rccm.200906-0896OC 19892860 PMC2822971

[64] Haldar P, Pavord ID, Shaw DE, Berry MA, Thomas M, Brightling CE, et al Cluster analysis and clinical asthma phenotypes. Am J Respir Crit Care Med. 2008;178(3):218–24. Epub 2008/05/16. 10.1164/rccm.200711-1754OC 200711-1754OC [pii]. 10.1164/rccm.200711-1754OC 18480428 PMC3992366

[65] Woodruff PG, Modrek B, Choy DF, Jia G, Abbas AR, Ellwanger A, et al T-helper type 2-driven inflammation defines major subphenotypes of asthma. Am J Respir Crit Care Med. 2009;180(5):388–95. Epub 2009/06/02. 10.1164/rccm.200903-0392OC 200903-0392OC [pii]. 10.1164/rccm.200903-0392OC 19483109 PMC2742757

[66] Emadi S, Clay D, Desterke C, Guerton B, Maquarre E, Charpentier A, et al IL-8 and its CXCR1 and CXCR2 receptors participate in the control of megakaryocytic proliferation, differentiation, and ploidy in myeloid metaplasia with myelofibrosis. Blood. 2005;105(2):464–73. Epub 2004/09/30. 10.1182/blood-2003-12-4415 2003-12-4415 [pii]. . 15454487 10.1182/blood-2003-12-4415

[67] Nair P, Gaga M, Zervas E, Alagha K, Hargreave FE, O'Byrne PM, et al Safety and efficacy of a CXCR2 antagonist in patients with severe asthma and sputum neutrophils: a randomized, placebo-controlled clinical trial. Clin Exp Allergy. 2012;42(7):1097–103. Epub 2012/06/19. 10.1111/j.1365-2222.2012.04014.x . 22702508

